# Roots and Nodules Response Differently to P Starvation in the Mediterranean-Type Legume *Virgilia divaricata*

**DOI:** 10.3389/fpls.2019.00073

**Published:** 2019-02-05

**Authors:** Gary G. Stevens, María A. Pérez-Fernández, Rafael J. L. Morcillo, Aleysia Kleinert, Paul Hills, D. Jacobus Brand, Emma T. Steenkamp, Alex J. Valentine

**Affiliations:** ^1^Department of Botany and Zoology, Stellenbosch University, Matieland, South Africa; ^2^Ecology Area, Universidad Pablo de Olavide, Sevilla, Spain; ^3^Shanghai Center for Plant Stress Biology, Chinese Academy of Sciences, Shanghai, China; ^4^Institute for Plant Biotechnology, Stellenbosch University, Matieland, South Africa; ^5^NMR Unit, Central Analytical Facility, Stellenbosch University, Matieland, South Africa; ^6^Department of Microbiology and Plant Pathology, Forestry and Agricultural Biotechnology Institute, University of Pretoria, Pretoria, South Africa

**Keywords:** legumes, nodules, low P, high P allocation of resources, biological nitrogen fixation, conservation strategies, phosphoenolpyruvate carboxylase, phosphate stress

## Abstract

*Virgilia divaricata* is a tree legume that grows in the Cape Floristic Region (CFA) in poor nutrient soils. A comparison between high and low phosphate growth conditions between roots and nodules was conducted and evaluated for the plants ability to cope under low phosphate stress conditions in *V. divaricata*. We proved that the plant copes with low phosphate stress through an increased allocation of resources, reliance on BNF and enhanced enzyme activity, especially PEPC. Nodules had a lower percentage decline in P compared to roots to uphold its metabolic functions. These strategies partly explain how *V. divaricata* can sustain growth despite LP conditions. Although the number of nodules declined with LP, their biomass remained unchanged in spite of a plant decline in dry weight. This is achieved via the high efficiency of BNF under P stress. During LP, nodules had a lower % decline at 34% compared to the roots at 88%. We attribute this behavior to P conservation strategies in LP nodules that imply an increase in a metabolic bypass that operates at the PEP branch point in glycolysis. The enhanced activities of nodule PEPC, MDH, and ME, whilst PK declines, suggests that under LP conditions an adenylate bypass was in operation either to synthesize more organic acids or to mediate pyruvate via a non-adenylate requiring metabolic route. Both possibilities represent a P-stress adaptation route and this is the first report of its kind for legume trees that are indigenous to low P, acid soils. Although BNF declined by a small percentage during LP, this P conservation was evident in the unchanged BNF efficiency per weight, and the increase in BNF efficiency per mol of P. It appears that legumes that are indigenous to acid soils, may be able to continue their reliance on BNF via increased allocation to nodules and also due to increase their efficiency for BNF on a P basis, owing to P-saving mechanisms such as the organic acid routes.

## Introduction

The Cape Floristic Region (CFR), found in the south western area of South Africa can be regarded as one of the highest P-impoverished regions of the world and simultaneously a Global Biodiverse Hotspot (Lambers and Shane, 2007). The CFR resembles a typical Mediterranean-type ecosystem usually characterized by sandstone-derived soils (Goldblatt and Manning, 2000), which are acidic, with insufficient nutrients (especially N and P) to sustain normal plant growth (Bordeleau and Prevost, 1994; Von Uexkull and Mutert, 1998; Grigg et al., 2008). In particular, legume species reliant on Biological Nitrogen Fixation (BNF) are highly dependant on P supply, more so than legumes growing on mineral N (Drevon and Hartwig, 1997). For legumes, P not only affects the formation of nodules (Israel, 1993), but limiting P also impacts negatively on the nitrogen fixation process (Schultze et al., 2006; Tsvetkova and Georgiev, 2007). The tree species, *V. divaricata* (Adamson), is a native legume to the CFR and it is distributed over a wide range of P-poor soils from the relatively richer forest margins to poorer Fynbos soils (Coetsee and Wigley, 2013). This implies that the indigenous species may have a range of mechanisms to adapt to variable soil P supply.

These mechanisms, therefore, have evolved adaptations to function optimally under limiting P conditions (Vance et al., 2003). Some strategies are aimed at conserving the use of P, whereas others are directed toward enhanced acquisition and uptake of P (Lajtha and Harrison, 1995; Horst et al., 2001; Vance et al., 2003). Adaptations that conserve the use of P involve a decrease in growth rate, increased growth per unit of P uptake, remobilization of internal P*i*, modification in C-metabolism that bypass P-requiring steps and alternative respiratory pathways (Schachtman et al., 1998; Plaxton and Carswell, 1999; Raghothama, 1999; Uhde-Stone et al., 2003a,b). In legumes, adaptations leading to enhanced P acquisition entail the expression of genes that result in the production of cluster roots. Cluster roots increase the root surface area. This enhances nodule efficiency for P utilization (Le Roux et al., 2008), root exudation of organic acids and acid phosphatase, as well as the induction of numerous transporters (Gilbert et al., 2000; Gilroy and Jones, 2000; Lynch and Brown, 2001; Neumann and Martinoia, 2002; Lamont, 2003; Uhde-Stone et al., 2003a; Vance et al., 2003).

The high sensitivity of legume plants, and indeed the N_2_- fixation process to environmental conditions such as acidic soils associated with P deficiency, may result in higher C costs (Mengel, 1994). This concurs with Le Roux et al. (2008), who showed that lupin nodules under P stress acted as stronger C sinks. Nodules are known to have a strong sink capacity for P assimilation during P starvation (Høgh-Jensen et al., 2002). The enhanced nodule cost for P utilization is considered to be an essential coping strategy during P stress (Le Roux et al., 2008). The C sink was found to be more pronounced in plants during symbiosis under low-P conditions (Mortimer et al., 2008). This was shown by a greater growth respiration of low-P plants than high-P plants (Mortimer et al., 2008). The sink effect was also evidenced by the higher photosynthetic rates of host plants (Mortimer et al., 2008). In the case of P stress, the most direct currency is P itself and growth parameters related to P accumulation (Koide and Kabir, 2000).

Physical changes to roots (adjustment of root architecture, root growth, root system composition and mycorrhizal infection) that takes place as a result of P limitations, are complemented by the exudation of a variety of organic compounds (carboxylate anions phenolics, caboxylates, amino acids enzymes, and other proteins), as well as inorganic compounds (protons, phosphate and nutrients) that into the rhizosphere aid the plants in the adaption for a particular nutrient stressed environment (Crowley and Rengel, 1999). The *Fabaceae* family develops cluster roots which are stimulated during phosphate stress. Not only do these species develop cluster roots, but also exude carboxylates which releases P from its bound form, making P more accessible for root uptake (Lambers and Shane, 2007). It was found that during P deficiency, plants exude carboxylates such as citrate, malate, malonate, acetate, fumerate, succinate, lactate, and oxalate in various concentrations (Rengel, 2002). White lupin exudes large amounts of carboxylates in the form of malic- and citric acid to the immediate soil surrounding to release P from its bound form in the soil. These excreted organic acids have the ability to chelate metal cations such as Al^3++^ and Ca^2+^ and immobilize P*i* in the soil, which results in higher P*i* concentrations in the soil up to 1000 fold (Gardner et al., 1983; Dinkelaker et al., 1989; Neumann et al., 2000). The production of these exudates are accomplished by the concerted action of a variety of enzymes, such as the Pyrophosphate (PP*i*), dependent phosphofructokinase (PP*i*-PFK), Phosphoenolpyruvate (PEP) phosphatase and Phosphoenolpyruvate Carboxylase (PEPC). Pyruvate, which is the precursor to many of these substances, can be generated in the cytosol and in the mitochondria. Cytosolic pyruvate is produced from PEP during the glycolytic conversion of ADP to ATP which is catalyzed by pyruvate kinase (PK) (Plaxton, 1996). It is suggested, that when plants experience P stress, that pyruvate synthesis from PEP via PK is restricted (Theodorou and Plaxton, 1993; Plaxton, 1996). However, pyruvate can also be generated from malate when plants make use of a “bypass” route especially during P-limitations. In this “bypass” route, PEP is hydrolyzed to Oxalacetic Acid (OAA) by PEPC and OAA is subsequently converted to malate by Malate Dehidrogenase (MDH). Mitochondrial Malic Enzyme (ME) converts malate into PEP (Plaxton, 1996).

In addition PEPC catalyzes the conversion of phosphoenolpyruvate and bicarbonate to oxaloacetate (OAA) and inorganic phosphate (Chollet et al., 1996). It is believed to play a pivotal role in carbon metabolism in symbiotic nodules of legume roots (Day and Copeland, 1991; Rawsthorne, 2002). The PEPC derived OAA can be converted to malate, via malate dehydrogenase. The generated malate can be fed into the mitochondrial tri-carboxylic acid cycle (TCA) for further metabolism, or metabolized to pyruvate via ME. PEPC plays a crucial role in the assimilation of atmospheric CO_2_ during C4 and CAM photosynthesis. PEPC has also been implicated to replenish the citric acid cycle intermediates when carbon skeletons are removed for other metabolic functions like nitrogen assimilation and amino acid biosynthesis when plants undergo P-stress The induction of PEPC during P-stress also results in elevated levels of organic acids such as malate and citrate in the rhizosphere (O’Leary et al., 2011) and dicarboxylic acids (Streeter, 1991; Tajima et al., 2015). Furthermore, N_2_ fixation comes with a high CO_2_ loss (Pate et al., 1993), which could account for more than 60% of the carbon allocated to the nodules (Voisin et al., 2007). Plants manage to reincorporate this CO_2_ as intermediates to the TCA cycle, and to fuel nodule metabolism, by the combined actions of carbonic anhydrase and PEPC (Vuorinen and Kaiser, 1997; Flemetakis et al., 2003).

Nuclear Magnetic Resonance (NMR) spectroscopy allows for the characterization of the metabolites in plant cells by coupling NMR with ^13^C stable isotope enrichment, as the ^12^C isotope is not NMR active. It can be used to determine the metabolite flux in plant cells making it suitable to establish the conditions and compartmentation of these metabolites in plant cells (Chang and Roberts, 1989; Gilbert et al., 2011). Photosynthetic CO_2_ fixation discriminates against ^13^C, therefore mainly the sodium bicarbonate-^13^C enriched solution supplied as feedstock will be metabolized by the plant. This ^13^C enrichment allows for the characterization of the resulting metabolic activities in plant cells by NMR. It was shown that this technique could be exploited to determine the metabolite flux in plant cells making it suitable to establish the conditions and compartmentation of these metabolites in plant cells (Chang and Roberts, 1989).

*Virgilia* is a small tree genus that includes two species *V. divaricata* (Adamson) and *V. oroboides* (P. J. Bergius, T. M. Salter). It is confined to the south-western and southern coastal regions of the CFR (Greinwald et al., 1989). Studies have been conducted on growth and adaptations of legume species native to Mediterranean-type fynbos ecosystems that occur on naturally acidic soils (Muofhe and Dakora, 1999; Spriggs and Dakora, 2008; Power et al., 2010; Kanu and Dakora, 2012). However, information on the physiology of N and P uptake, efficiency and utilization in legume trees in fynbos soils is largely unknown. Although the CFR has a high legume diversity found on the P-poor soils (Goldblatt and Manning, 2000), not much is known about the functional mechanisms which underpin N nutrition within the nodules of these indigenous legumes. The adaptation to P stress may involve a variety of morphological and biochemical mechanisms that are related to enhancing acquisition of soil P, recycling of internal P*i* and conserving available internal P. Recent work from our group has shown that *Virgilia* uses a variety of strategies to adapt to low P conditions. Magadlela et al. (2014) compared two species within the genus *Virgilia*, and demonstrated that *V. divaricata* maintained a high efficiency of BNF, owing to a greater allocation of biomass toward nodules during P deficiency. Vardien et al. (2014) showed that nodules have a high functional plasticity during variable P supply, by recycling organic P via acid phosphatase enzymes and redistributing Fe within the nodule. In the present study we investigated the root system engagement of a non-P requiring metabolic bypass and its implications to nodule efficiency of the indigenous legume *V. divaricata* during variable P supply. We aimed at gathering a better understanding of how nodules manage to sustain their functioning during P-stress. To that end we investigated how PEPC-derived C is metabolized into amino acids and downstream organic acids of P-deficient nodules, using ^13^C NMR spectroscopy. We hypothesized that plants of *V. divaricata* grown in P-poor soils, have evolved adaptive mechanism which conserve internal P and are designed for maintaining nodule function during P deficiency.

## Materials and Methods

### Plant Growth

Sterile seeds of *V. divaricata* (Silverhill Seeds, Kenilworth, Cape Town, South Africa) were pre-treated with smoke water and water at 50°C for 5 h, to enhance their germination (Soos et al., 2009). Seeds were then allowed to germinate in sterile filter sand in seed-trays placed in a north facing glass house under natural light conditions. Plants were exposed to a photo and thermo period of 10 h sunlight at 25°C and 14 h in darkness at 15°C. Seedlings were transferred to pots with sterile filtered sand after 2 weeks of growth, when the first true leaves had emerged. At this stage, seedlings were harvested and dried and used as the first harvest, from which to calculate growth rates. All plants were inoculated with the nodule forming *Burkholderia phytofirmans*. Inoculation treatments consisted of 500 μl of growth phase broth cultured inoculant at about 1.106 cells ml^−1^. Plants were divided into two groups, i.e., low (5 μM) –and high (500 μM) phosphate according to the Long Ashton nutrient treatment. Plants received the respective treatments twice per week and were allowed to grow for 8 weeks before harvest. Seedlings were divided into leaves, stems roots and nodules which were, respectively, weighed for their fresh weights. Nodules were kept in Eppendorfs at −80°C until analyzed. The leaves stems and roots were dried in a 50°C until constant weight prior to analysis.

### Protein Extraction

Plant material, either roots or nodules, were ground to a fine powder in liquid nitrogen. Proteins from roots and nodules were extracted according to the methods used by Ocaña et al. (1996) and was modified to an extent that 0.5 g of tissue was extracted in 2 ml of extraction buffer consisting of 100 mM Tris–HCl (pH 7.8), 1 mM Ethylenediaminetetraacetic acid (EDTA), 5 mM dithiothreitol (DTT), 20%(v/v)ethylene glycol, plus 2%(m/v) insoluble polyvinylpolypyrrolidone (PVPP) and one Complete Protease Inhibitor Cocktail tablet (Roche Diagnostics, Randburg, South Africa) per 50 ml of buffer. The protein concentration was determined by the NanoDrop Lite Spectrophotometer (Thermo Scientific) where the extraction buffer was used as standard.

### Enzyme Assays

All enzyme assays were carried out at 25°C in a multi-well plate reader at a wavelength of 340 nm. All reactions contained 30 μl of the crude extraction mixture in a final volume of 250 μl.

### Phosphoenolpyruvate Carboxylase

Phosphoenolpyruvate carboxylase activity was coupled with the NADH-malate dehydrogenase and measuring NADH oxidation at 25°C by monitoring NADH oxidation at 340 nm. The standard assay mixture (pH 8.5) contained 100 mM Tris (pH 8.5), 5 mM MgCl_2_, 5 mM NaHCO_3_, 4 mM PEP, 0.20 mM NADH, and 5 units of MDH (Ocaña et al., 1996). Measurement was carried out against 9 blanks without PEP. Two measurements were taken for each treatment. All reactions were performed in triplicate.

### Pyruvate Kinase

Pyruvate kinase was assayed at room temperature (22–24°) by recording at 340 nm the oxidation of NADH. The incubation mixture contained 75 mM Tris–HCl (pH 7.0), 5 mM MgCl_2_, 20 mM KCl, 1 mM ADP, 3 mM PEP, 0.18 mM NADH and 3 units of lactate dehydrogenase (McCloud et al., 2001), and 2 units of lactate dehydrogenase in a total volume of 1 ml. The blanks consisted of the buffer without ADP.

### Malic Enzyme

Malic enzyme activity was assayed by measuring the increase in 340 nm due to the formation of NADH or NADPH. Standard reaction mixture contained 80 mm Tris–HCl (pH 7.5), 2 mm MnCl2, 1 mm malate and 0.4 mm NADP or NAD^+^ (Appels and Haaker, 1988).

### NADH-Malate Dehydrogenase

The MDH activity was measured in 25 mM KH_2_PO_4_, 0.2 mM NADH, 0.4 mM oxaloacetate (OAA), pH 7.5 (Appels and Haaker, 1988). 25 mM KH_2_PO_4_, 0.2 mM NADH, 0.4 mM OAA the rate of disappearance of NADH was monitored at 340 nm before and after addition of oxaloacetate. The former rate served as a measurement of background NADH oxidation which was subtracted from the rate of oxaloacetate-dependent activity. Initial reaction rates have been shown to be proportional to the concentration of enzyme under the conditions used in these experiments. The assay system for measuring the oxidation of malate by NAD^+^, catalyzed by malate dehydrogenase, involves the reaction of oxaloacetate with L-glutamate in a subsequent reaction catalyzed by glutamate-oxaloacetate transaminase. The assay system contained 50 mM Tris/HCl, 40 mM L-glutamate, 0.8 mM NAD^+^, 4.0 U/ml glutamate oxaloacetate transaminase and 100 mM L-malate, pH 8.0. The reaction rates were measured from the appearance of NADH absorbance at 340 nm. The amount of NADH and oxalacetate formed in the oxidation of malate was stoichiometric 11 with the amount of malate and NAD^+^. Initial reaction rates have been shown to be proportional to the concentration of enzyme under the conditions used (Appels and Haaker, 1988).

### Citric- and Malic Acid Determination

Citric- and malic acid content for HP and LP nodules and roots were determined using a photometric analyzer (Arena 20XT, Thermo Electron Oy, Finland), which measures the amount of product formed after an enzymatic reaction. The reactions were performed in triplicate. The pH of the samples was adjusted to between 8 and 10 at room temperature. Reactions inside the instrument were performed at 37°C. Citrate and malic acid concentrations were determined by the enzymatic conversion of citrate and malate. In the process, NADH is oxidized which is stoichiometric to the amount of citrate and malate, respectively. NADH is then photometrically determined at 340 nm.

### Phosphate Determination

Phosphate analysis was performed on HP and LP samples of roots and nodules For the determination of total P, approximately 0.25 g of the sample material was digested in 7 ml HNO_3_ in a Mars CEM microwave digester, then diluted into 50 ml deionized water. P was measured on a Thermo ICAP 6300 ICP-AES after calibration of the instrument with NIST-traceable standards.

### Isotope Analysis

Analyses of δ15N were done at the Archeometry Department at the University of Cape Town, where the isotopic ratio of δ^15^N was calculated as δ = 1000‰ (Rsample/Rstandard). *R* refers to the molar ratio of the heavier to the lighter isotope of the samples. Standards were similar to those as described by Farquhar et al. (1989). Combustion of the samples were performed in a CHN analyzer (Fisons NA 1500, Series 2, Fisons instruments SpA, Milan, Italy) and the δ^15^*N* values for the nitrogen gas released were determined on a Finnigan Matt 252 mass spectrometer (Finnigan MAT GmbH, Bremen, Germany), which was connected to a CHN analyzer by a Finnigan MAT Conflo control unit. The sample values were corrected by the use of three standards. Two in-house standards (Merck Gel and Nasturtium) were used and the third was the IAEA (International Atomic Energy Agency) standard (NH_4_)_2_SO_4_. The percentage of nitrogen derive from atmospheric fixation (%NDFA) was calculated according to Shearer and Kohl (1986), where:

%NDFA=100((δ15Nreference plant−δ15Nlegume)/(δ15N reference plant−B).

Wheat (*Triticum aestivum*) was used as reference plant which was grown under the same glasshouse conditions as the legume. The B-value (which was determined as −0.71‰.) refers to the δ^15^N natural abundance of the N derived from biological N-fixation of the above-ground tissue of *V. divaricata*, grown in an N-free solution.

### ^13^C Enrichment

In order to investigate the metabolism of belowground incorporation and metabolism of ^13^C labeled bicarbonate in roots and nodules, NMR spectroscopy was used by coupling NMR with ^13^C stable isotope enrichment at the root-zone level. Plants of *V. divaricata*, were grown in sterile sand culture under two levels of P supply, low (5 μM) P and high (500 μM) P nutrition. At 2 months of age, both the low P and high P plants were supplied with a sodium bicarbonate-^13^C labeled solution in the pots. The pots were sealed and contained a CO_2_ trap, to prevent ^13^C leakage to the atmosphere. The experimental procedure was as follows. Plants of both treatments were enriched with ^13^C at the root-zone level, by watering them with a 300 ml solution of sodium bicarbonate-^13^C labeled (pH 6.8) (Sigma Aldrich Cat # 372382-1G, 99 atom % ^13^C) (0.215 g/L). A solution of KOH (250 mM) was placed in trays at the bottom of the pots to absorb CO_2_ which could escape through soil. Lids, designed with a special opening to cover the sand and below-ground organs, but allow the shoots to be exposed to the atmosphere, were placed on pots immediately after 300 ml of NaH^13^CO_3_ solution was fed. These lids were made completely airtight around the pots. In addition, CO_2_ traps were inserted into the lids, to prevent any NaH^13^CO_3_ from escaping to the atmosphere. These traps consisted of Soda Lime in 5 ml pipette tips and were inserted in the head space between the lid and soil in pot. The insertion points of traps into the lids were sealed off and made air tight. The run-off volumes of the NaH_13_CO_3_ were collected and measured. Pots were then placed on clean trays with fresh KOH. Plants were harvested at 1 h and 2 h intervals after feeding of NaH^13^CO_3_. All metabolic processes were stopped by quenching nodulated roots in liquid N_2_.

### ^13^C NMR

Sample preparation was done as described in Gout et al. (1993). Briefly, 4.5 g of roots and nodules were frozen in liquid N_2_ and ground to a powder in 1 ml of 70% (v/v) perchloric acid. The frozen powder was allowed to thaw at −10°C. The thick slurry was the centrifuged at 15000 rpm for 10 min and the supernatant was then neutralized with 2M KHCO_3_ to pH 5. The supernatant was then centrifuged at 10000 rpm for 10 min to remove KClO_4_ and then lyophylised and stored in liquid N_2_. The lyophilized sample was re-dissolved in 2.5 ml water which contained 10% (v/v) 2H_2_O. The solution was neutralized to pH 7.5, buffered with HEPES and CDTA (50–100 μM) was added to chelate divalent cations. Their respective ^13^C NMR spectra was recorded at 25°C dissolved in D_2_O on a Agilent Inova 600MHz spectrometer utilizing the default pulse sequence parameters in the VnmrJ 4.2 instrument software package.

### Calculations

#### Specific N Absorption Rate

Specific N absorption rate (SNAR) (mg N g^−1^ root DW d^−1^) is the net N absorption rate per unit root DW as outline in Nielson et al. (2001), and it was calculated as:

SNAR=[(M2−M1/t2−t1)]×[(loge R2−loge R1)/(R2−R1)]

Where *M* is the N content per plant, *t* is the time elapsed between two harvests and *R* is the root DW.

#### Belowground Allocation

Belowground allocation refers to the fraction of new biomass partitioned into new roots and nodules over the given growth period. The calculations were done according to Bazzaz and Grace (1997) as follows:

df/dt=RGR (∂−Br/Bt)

Where RGR is the relative growth rate (mg.g^−1^.day^−1^) and ∂ is the fraction of new biomass gained during the growth period. Br/Bt is the root weight ratio, based on total plant biomass (Bt) and root biomass (Br).

### Statistical Analysis

The effects of the factors and their interactions were tested with an analysis of variance (ANOVA) (KaleidaGraph, Synergy Software, PA, United States). Where the ANOVA revealed significant differences between treatments, the means (6–8) were separated using *post hoc* Tukey’s LSD (SuperANOVA for Macintosh, Abacus Concepts, United States) (*P* ≤ 0.05).

## Results

### Biomass

**FIGURE 1:**
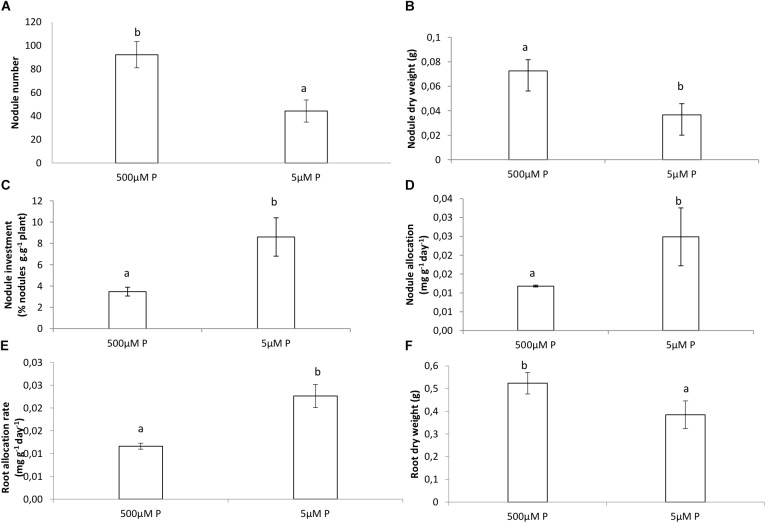
**(A)** Amount of nodules on roots, **(B)** dry weight of nodules, **(C)** nodule % of plant dry weight, **(D)** nodule allocation rate, **(E)** root allocation rate, **(F)** root dry weight of *Virgilia divaricata* grown under high phosphate (500 μM P) and low phosphate (5 μM P) conditions. Values of four replicates are presented as means ± SE. Different letters indicate significant differences between treatments (*P* ≤ 0.05).

**FIGURE 2:**
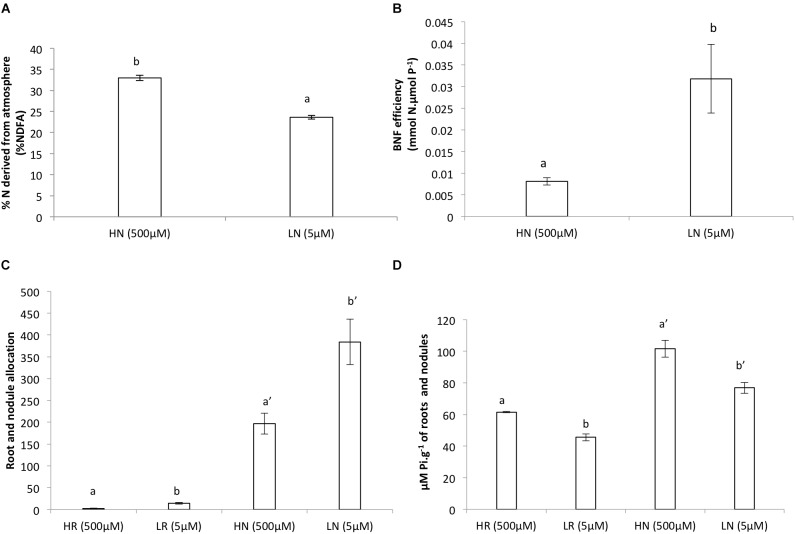
**(A)** Biological nitrogen fixation (BNF), **(B)** efficiency of biological nitrogen fixation (BNF) per unit of metabolic P in nodules, **(C)** root and nodule allocation, **(D)** internal P*i* of roots and nodules of *V. divaricata*, grown under high phosphate (500 μM P) and low phosphate (5 μM P) conditions. HN (high P nodules), LN (low P nodules, HR (high P roots), and LR (low P roots are compared). Values of four replicates are presented as means ± SE. Different letters indicate significant differences between treatments (*P* ≤ 0.05).

The dry weight (DW) of the roots and nodules was significantly much lower in the LP treatment compared to the HP treatment (Figure 1A,B,F). The relative growth rate for roots was much higher for the LP treatment and slightly higher in nodules of the LP treatment compared to the HP treatment (Figure 1D,E). However, nodulation in the LP treatment was much lower and more than twice the amount of nodules were formed in the HP treatment (Figure 1A,B). The plants allocated more of their resources to nodules and to roots in LP than in HP (Figure 1C,E). The allocation of resources for both treatments remained almost similar in the nodules (Figure 2C). Internal Pi of roots and nodules was significantly lower in the LP treatments (Figure 2D). However, the nodules were more efficient in BNF per dry weight in the LP nodules and there was a decline in %NDFA in the same nodules (Figure 2A,B).

### Biological Nitrogen Fixation

During low P supply, there was a decline in BNF (%NDFA) compared to the HP supply (Figure 2A). However, in spite of the decline in BNF, the efficiency of BNF per unit P was higher in the LP treatment compared to the HP (Figure 2B).

### Protein and Enzyme Assays

**FIGURE 3:**
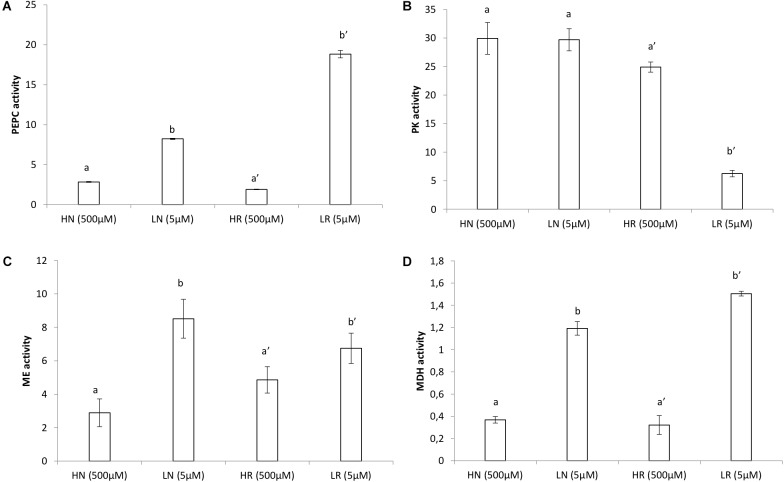
Enzyme activities (μmol.min^−1^.g^−1^FW) in roots and nodules of *V. divaricata* grown under high phosphate (500 μM) and low phosphate (5 μM) conditions **(A)** PEPC, **(B)** PK, **(C)** ME, **(D)** MDH. HN (high P nodules), LN (low P nodules, HR (high P roots), and LR (low P roots are compared). Values of four replicates are presented as means ± SE. Different letters indicate significant differences between treatments (*P* ≤ 0.05).

Measurements of PEPC, MDH, and ME in plants grown in LP rended higher values than those obtained in plants grown in HP (Figure 3A,C,D). The highest PEPC (Figure 3A) activity was in LP roots which was more than four times higher compared to HR roots. PEPC activity in nodules was double compared to HP nodules (Figure 3A). PK activity (Figure 3B) was higher in the HP conditions compared to the LP conditions. Almost similar PK activity was found for HP in roots and HP in nodules. The PK activity in LP nodules was slightly less than that in HP nodules. PK activity was five times higher in HP roots compared to LP roots (Figure 3B). The highest ME activity (Figure 3C) was obtained in LP nodules which was double of that in HP nodules. The greatest activity was also found in LP nodules compared to HP nodules (Figure 3C). MDH activity per fresh weight was five times higher in LP nodules compared to HP nodules and was more than double in LP roots compared to HP roots (Figure 3D).

### Organic Acids

**FIGURE 4:**
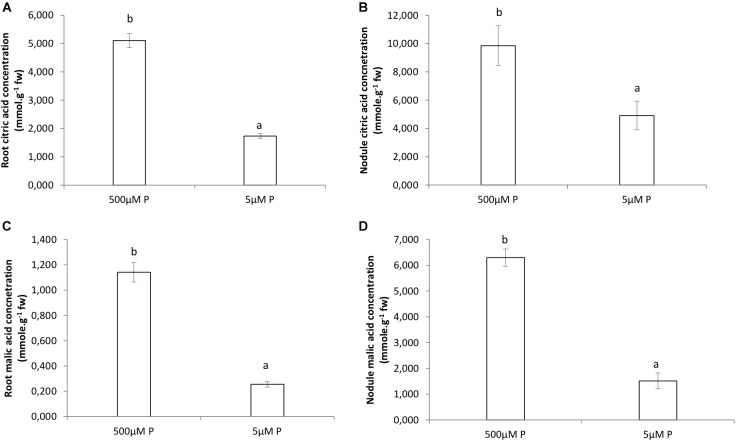
Organic acids concentrations (mg.mol.g^−1^ FW) by GCMS analysis in roots and nodules of *V. divaricata*, grown under high phosphate (500 μM P) and low phosphate (5 μM P) conditions. Citric acid concentration in **(A)** roots, **(B)** nodules. Malic acid concentration in **(C)** roots **(D)**, nodules. Values of four replicates are presented as means ± SE. Different letters indicate significant differences between treatments (*P* ≤ 0.05).

Organic acids were higher in roots and nodules receiving the HP treatment (Figure 4). The citric acid concentration in HP roots was almost fivefold the amount compared to LP roots (Figure 4A). The amount of citric acid found in nodules at HP was double of that in LP (Figure 4B). The malic acid concentration in HP roots was one order of magnitude greater than the amount found in LP roots (Figure 4C). The amount of malic acid found in nodules at HP was sixfold greater of that in LP (Figure 4C).

### Inorganic P Data

**FIGURE 5:**
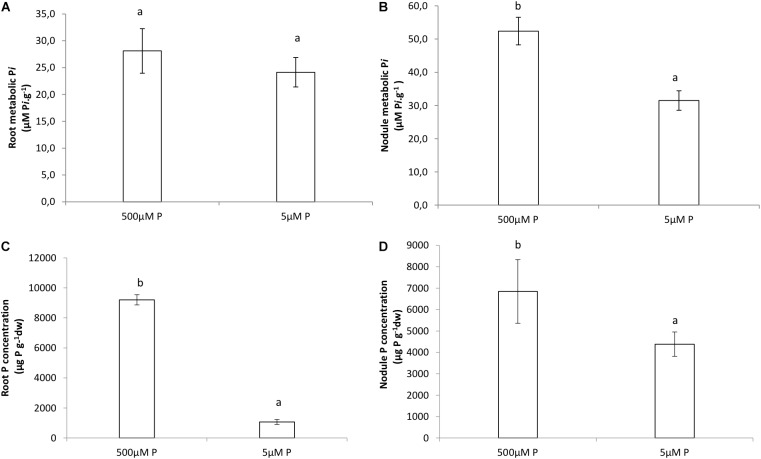
Internal P*i* (μmol P*i*.g^−1^) of **(A)** roots, **(B)** nodules. Phosphate concentration (mg.kg^−1^) in **(C)** roots, **(D)** nodules of *V. divaricata* grown under high phosphate (500 μM P) and low phosphate (5 μM P) conditions. Values of four replicates are presented as means ± SE. Different letters indicate significant differences between treatments (*P* ≤ 0.05).

Higher internal P*i* values were obtained in the HP treatment for both roots and nodules, although we only found significant differences between HP and LP in the concentrations of nodules (Figure 5A,B). Phosphate concentration was significantly greater in HP treatments than in LP for both roots and nodules (Figure 5C,D).

### NMR

An array of ^13^C NMR spectra was recorded for each individual plant extract. The carbonyl carbon of the different organic acids each appear at a unique chemical shift area, between 175 and 181 ppm, in the respective spectra (Supplementary Figures S1–S4 for partial spectra and Supplementary Figures S5, S6 for full spectra). The unique carbonyl chemical shift of each organic acid were established by running commercial reference solutions of these organic acids (solubilized in D_2_O at pH 7.5), under the same conditions as the extract samples. These two signals were consequently assigned to the organic acids malate and citrate, respectively. Much higher (one order of magnitude) relative malate concentrations were found compared to citrate (Figure 7). Incorporation of ^13^C was very noticeable during the first hour with higher relative concentrations and a sharp decline in relative concentration after 2 h of exposure to ^13^C, especially citrate (Supplementary Figures S1, S2).

**FIGURE 6:**
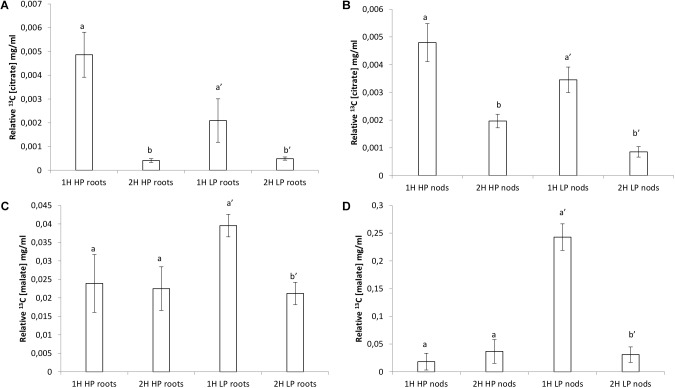
Relative organic acid concentrations (mg.ml^−1^) found by ^13^C NMR analysis in roots and nodules of *V. divaricata*, grown under high phosphate (500 μM P) and low phosphate (5 μM P) conditions **(A)** root malate, **(B)** nodule malate, **(C)** root citrate, **(D)** nodules citrate.

**FIGURE 7:**
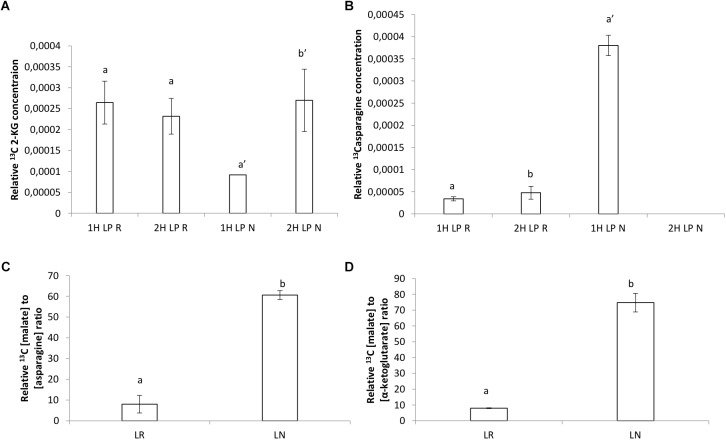
**(A)** Relative ^13^C α-ketoglutarate concentration after 1 h, **(B)** relative ^13^C asparagine concentration after 1 h, **(C)** relative ^13^C malate converted to asparagine concentration, **(D)** relative ^13^C malate converted to α-ketoglutarate concentration (mg.ml^−1^)found by ^13^C NMR analysis in roots and nodules of *V. divaricata*, grown under low phosphate (LP) (5 μM P) conditions.

Citrate levels were significantly higher in HP and LP roots and nodules after 1 h exposure (Figure 6A,B). Malate levels remained almost unchanged in HP conditions, however, a significant decline was observed in LP conditions in roots after 2 h of exposure (Figure 6C). Malate in nodules remained constant in all treatments without a strong increase after 1 h in LP (Figure 6D and Supplementary Figure S3). The presence of a keto-group (at 200–220 ppm) could also be observed in a few of the LP root and nodule spectra, which can be assigned to that of 2-ketoglutarate (Supplementary Figure S4). The peak at 161 ppm, which was present in all the samples (except in the control sample) can be assigned to ^13^C bicarbonate which was present in the perfusion medium (Gout et al., 1993). Samples concentrations were corrected by dividing peak areas into the ^13^C bicarbonate peak area at 161 ppm. Significantly greater concentration of 2-ketoglutarate was recorded in LP nodules after 2 H (Figure 7A). Levels of asparagine were very low both in roots and shoots except for the significantly greater concentration measured in LP nodules after 1 h (Figure 7B). The relative malate converted to asparagine was significantly greater in the nodules at LP (Figure 7C) and the same can be said about the relative malate converted to α-ketoglutarate (Figure 7D).

## Discussion

During P deficiency, *V. divaricata* nodules experienced less P*i* stress than roots, due to increased metabolic P conservation reactions during organic acid synthesis. Although the BNF declined, the high efficiency of BNF may be underpinned by these altered P conservation pathways and enhanced resource allocation during growth.

In legumes, Biological Nitrogen Fixation (BNF) is highly dependent on phosphate supply, which affects nodule formation (Valentine et al., 2017) as well as the nitrogen fixation process (Schultze et al., 2006; Tsvetkova and Georgiev, 2007). However, certain types of legumes are adapted to fix N_2_ efficiently in P-impoverished environment. Particularly, *Virgilia divaricata*, a native legume tree to the Cape Floristic Region of South Africa, with high potential as precursor of Fynbos forests, has evolved to grow under low phosphate stress conditions, through previously unknown mechanisms. Our results indicate that belowground organs, roots and nodules, had a higher resource allocation under LP conditions as a consequence of their potential for greater contribution to mineral nutrition. This concurs with other species during P stress (Almeida et al., 2000) and also with legumes from nutrient poor ecosystems (Magadlela et al., 2014; Vardien et al., 2014), which can be interpreted as a strategy of legumes to adapt to scarce nutrient supply (Araújo et al., 2015).

Although there was a decline in the number of nodules in the LP treatment, the unchanged total nodule mass indicates that plants allocate more resources to existing nodules, thus increasing and maintain their efficiencies during LP conditions. This is supported by the efficiency of nodule functioning (compared to roots), under LP conditions, as reflected in the maintenance or proportionally lower decline of P levels during P stress. This lower decline in P concentration in nodules may also be attributed to the fact that nodules are P scavengers acquiring this nutrient mostly from roots, as reflected in the higher amounts of P and P*i* in nodules compares to roots in the LP-treatment, oriented to maintain their functioning (Jakobsen, 1985; Israel, 1993; Le Roux et al., 2006). Similar findings also indicated that nodule growth and functioning of this species is not limited by P-deficiency in white clover (Almeida et al., 2000) and also concurs with findings for *Medicago truncatula* where the P concentration in nodules seems to be unaffected as most of the P was allocated to the nodules (Sulieman et al., 2010). All these findings support the idea that *V. divaricata* is able to store P in the underground organs as an adaptation to the naturally low P environment where it naturally occurs.

In spite of the decline in BNF, there was an increase in BNF efficiency per mole P. This increase in BNF efficiency during low P supply, suggests that nodules attenuate their BNF capacity, despite low P conditions. It has been suggested that the decrease in nitrogen fixation in P stressed plants, should be viewed in correlation with whole plant growth, while specific nitrogenase activity is still maintained (Schultze, 2003). This idea is supported by various experimental evidences that it is the plant N status which regulates nitrogen fixation rates (Schultze, 2003). In addition, it appears that *V. divaricata* might also be able to shift its acquisition of N from BNF to soil N acquisition. This is reflected in higher mineral N uptake of nodulated roots as evidenced by the increase in specific root system N acquisition rate during P deficiency when BNF declines. This is in contrast to findings by Vardien et al. (2014), where roots showed a decline in mineral N uptake during P deficiency, compared to the current increase of mineral N in the nodulated root system. These differences may reside in the fact that in the current system, both roots and nodules may have contributed to mineral N uptake from soil. It is known that in a nodulated root system, both roots (Magadlela et al., 2014) and nodules can seperately acquire and assimilate mineral/soil N (Becana and Sprent, 1987) within a nodulated root system, which confers additional advantages to the plant growing in extremely poor soils.

Soil derived N is usually taken up in the form of NO_3_^−^ (Lambers and Shane, 2007) and it might be that the roots increase their contribution to acquire N under P limitation. Although root nitrate uptake by roots could be beneficial to plant metabolism, it could also impact negatively on BNF as it might inhibit nitrogenase activity in legume plant nodules. It was shown that nitrate impacts negatively on *Rhizobium*-infection as well as on the ratio of the nodule dry mass to the whole plant mass (Luciñski et al., 2002). As BNF is a costly process, it may be more beneficial for legumes from low nutrient ecosystems to take up N via its roots and to reduce energetically costly BNF (Magadlela et al., 2015). Similar trends were also observed in white clover where N concentration was unaffected by P deficiency. It was found in white clover that N_2_ fixation increased strongly under P deficiency and that approximately 30% of N was assimilated due to N_2_ fixation (Almeida et al., 2000). Similar to the BNF in white clover, we calculated that the nodules in our LP treatment experiment derived approximately 32% of the N from the atmosphere. The approximate 68% of N might be from soil uptake (whether directly by the nodules or via roots), as the plants were fed with nutrient solution containing NH_4_NO_3_. In support of the above, we obtained higher specific nitrogen acquisition rate values for naked roots compared to nodulated roots, irrespective the treatment). This could be an indication that the plant would rather utilize soil N instead of utilizing the costly BNF route, which could justify abovementioned findings.

In spite of variable P supply, the unchanged N levels are also reflected in the elevated levels of all major amino acids found in the nodules of the LP treatment compared to the nodules of the HP treatment, and both the treatments for roots. A similar trend was seen in P deficient white clover (Almeida et al., 2000) and *Medicago truncatula* (Sulieman et al., 2010), where elevated levels of all major amino acids were founds, especially asparagine. It appears that aspartate, which serves as a precursor to asparagine, also plays a key role in the maintenance of these processes, as it was the predominant amino acid found in this study (Maxwell et al., 1984; King et al., 1986; Rosendahl et al., 1990). Asparagine, which is usually found in elevated levels during low P conditions, can act as a possible N-feedback regulator to the nodules during P-stress, as it flows from the shoots to the nodules and conveys the message of the shoot nitrogen status to the nodules and modulates their activity according to nutrient status of the plant (Sulieman et al., 2010). In this way the nitrogenase activity can be regulated by asparagine and this trend is also similar in other legumes and non-legumes plants under stress (Steward and Larher, 1980; Lea et al., 2007).

The key to these generated amino acids and other metabolic products during P stress might lie in the operation of the non-adenylated PEPC bypass route. Various studies have implicated this non-adenylated PEPC-bypass route to increase the PEP metabolism during P deficiency (Duff et al., 1989; Theodorou and Plaxton, 1993). Those studies have also found that the PEPC-activity may lead to an increase malate production. Malate could serve as C fuel in bacteroides, which is generated by the combined action of CA, PEPC and MDH (Vance and Heichel, 1991). In addition, malate can be transformed into OAA through MDH and serves as C skeleton to generate Asparagine, which serves as the principle N export compound in temperate legumes (Schultze et al., 2006). The higher accumulation of malate in the nodules compared to the roots (irrespective the treatment), might implicate its role as C fuel for nodules to sustain nodule activity. Similar findings were also observed in white lupin, where higher malate concentrations were also found in nodules compared to roots (Schuller and Werner, 1993). In addition to its role as fuel for nodules, malate as well as citrate can be excreted by roots to chelate metal cations such as Fe^3+^, Fe^2+^, Al^3+^, and Ca^2+^ and in the process it release P from these cations, especially during low P conditions (Neumann and Römheld, 1999). The larger amount of citrate accumulation in roots compared to nodules may be an indication that *V. divaricata* also follows this trend to acquire P.

Although a combined action of all three enzymes (CA, PEPC, and MDH) is needed to generate organic acids for bacterial fuel and for exudation, literature highlights Class1 PEPC as playing a crucial role in the anaplerotic replenishment of tricarboxylic acid cycle intermediates where carbon skeletons are removed for other metabolic functions like nitrogen assimilation and amino acid biosynthesis especially during P-deficiency (Uhde-Stone et al., 2003b; Vance et al., 2003; Shane et al., 2004; O’Leary et al., 2011). When an extremely low level of P in the plant is reached, PEPC (in conjunction with MDH and ME) can theoretically function as a glycolytic enzyme by indirectly bypassing the conventional ADP dependent PK reaction to facilitate continued pyruvate supply to the TCA cycle. In the process, P*i* is also generated and recycled in the P-starved cells (Nagano et al., 1994; Plaxton and Carswell, 1999). *In vitro* root-MDH activity (LP treatment) appears to be the only enzyme to show higher activity over that of nodule-MDH activity. A direct result of this elevated LP root MDH activity might have been the export of malate to nodules which gave rise to the higher malate concentration in nodules, compared to roots). These findings give an indication that P deficiency may impact negatively on the root’s metabolic processes resulting in the lower biomass obtained for roots compared to the apparent unaffected nodule metabolism, resulting in an increase in biomass for nodules under P-stress.

## Conclusion

For legumes such as *V. divaricata* growing in P-poor soils, the continued reliance on BNF is underpinned by several key nodule adaptations. During P deficiency the nodules of *V. divaricata* have an increased allocation of resources and P-conservation mechanisms, which improve the efficiency of nodule BNF. These adaptations form the key to the plant’s ability to adapt to poor P environments and thus sustaining its reliance on BNF.

## Author Contributions

All authors have equally contributed to the manuscript, from its design to finalized the manuscript.

## Conflict of Interest Statement

The authors declare that the research was conducted in the absence of any commercial or financial relationships that could be construed as a potential conflict of interest.

## Supplementary Material

FIGURE S1 A section of the ^13^C spectra for **(a)** roots after 1 h, **(b)** roots after 2 h grown under high phosphate (500 μM P) conditions of *V. divaricata.*

FIGURE S2 A section of the ^13^C spectra of **(a)** roots after 1 h, **(b)** roots after 2 h grown under low phosphate (5 μM P) conditions of *V. divaricata.*

FIGURE S3 A section of the ^13^C spectra of **(a)** nodules after 1 h, **(b)** nodules after 2 h grown under high phosphate (500 μM P) conditions of *V. divaricata.*

FIGURE S4 A section of the ^13^C spectra of **(a)** nodules after 1 h, **(b)** nodules after 2 h grown under low phosphate (5 μM P) conditions of *V. divaricata.*

FIGURE S5 A sample of the full ^13^C spectra of roots after 1 h, from plants grown under high phosphate (500 μM P) conditions of *V. divaricata.*

FIGURE S6 A sample of the full ^13^C spectra of nodules after 1 h, from plants grown under high phosphate (500 μM P) conditions of *V. divaricata.*

## Abbreviations

BNF: biological nitrogen fixation
CFR: Cape Floristic Region
DW: dry weight
FW: fresh weight
HP: high phosphate
LP: low phosphate
MDH: malate dehydrogenase
ME: malic enzyme
NDFA: nitrogen derived from atmosphere
PEP: phosphoenol pyruvate
PEPC: phosphoenolpyruvate carboxylase
P*i*: inorganic phosphate
PK: pyruvate kinase
PP*i*: pyrophosphate
SNAR: specific nitrogen acquisition rate.
