# Supplementary figures and images for: Roots and Nodules Response Differently to P Starvation in the Mediterranean-Type Legume Virgilia divaricata
Source: Front Plant Sci. 2019 Feb 5;10:73. doi: 10.3389/fpls.2019.00073 (PMC6370976; doi:10.3389/fpls.2019.00073)

(a)

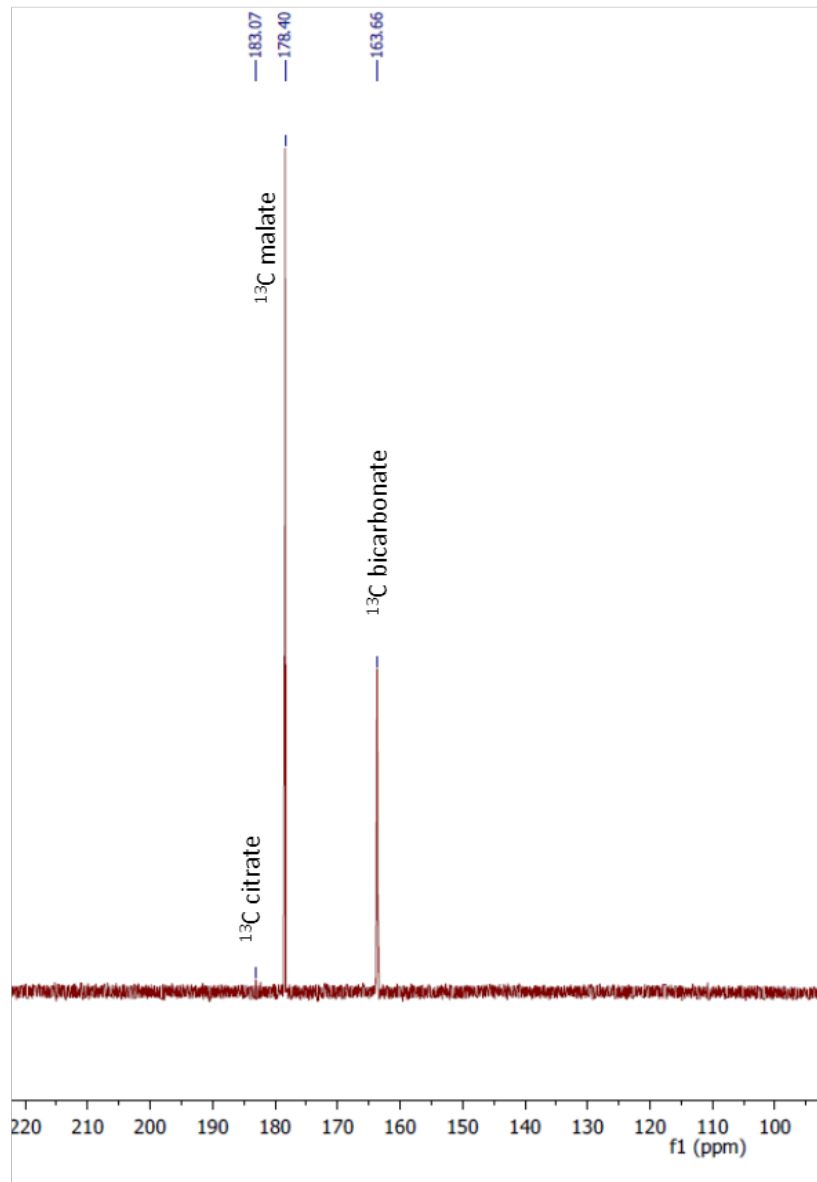

(b)

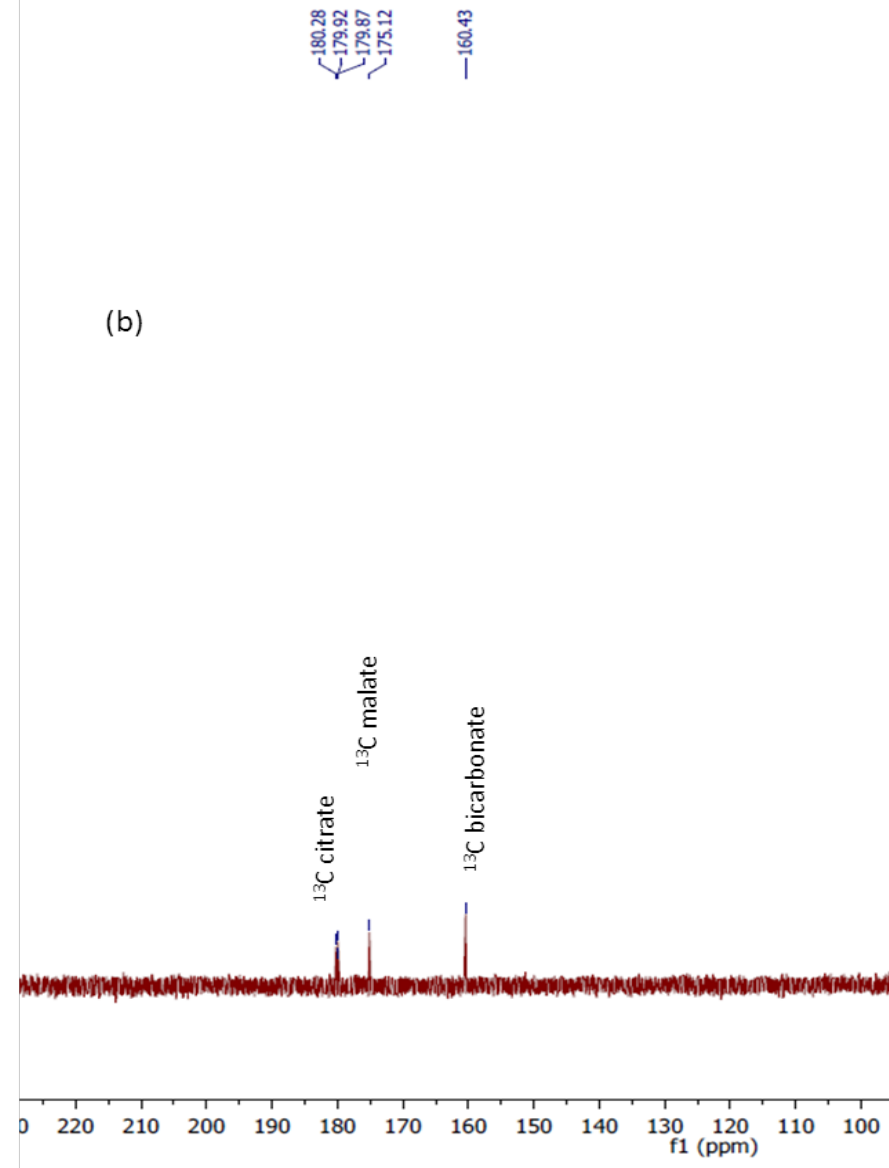

Supplement: FIGURE S1 — A section of the 13C spectra for (a) roots after 1 h, (b) roots after 2 h grown under high phosphate (500 μM P) conditions of V. divaricata. [file Data_Sheet_1.PDF]

(a)

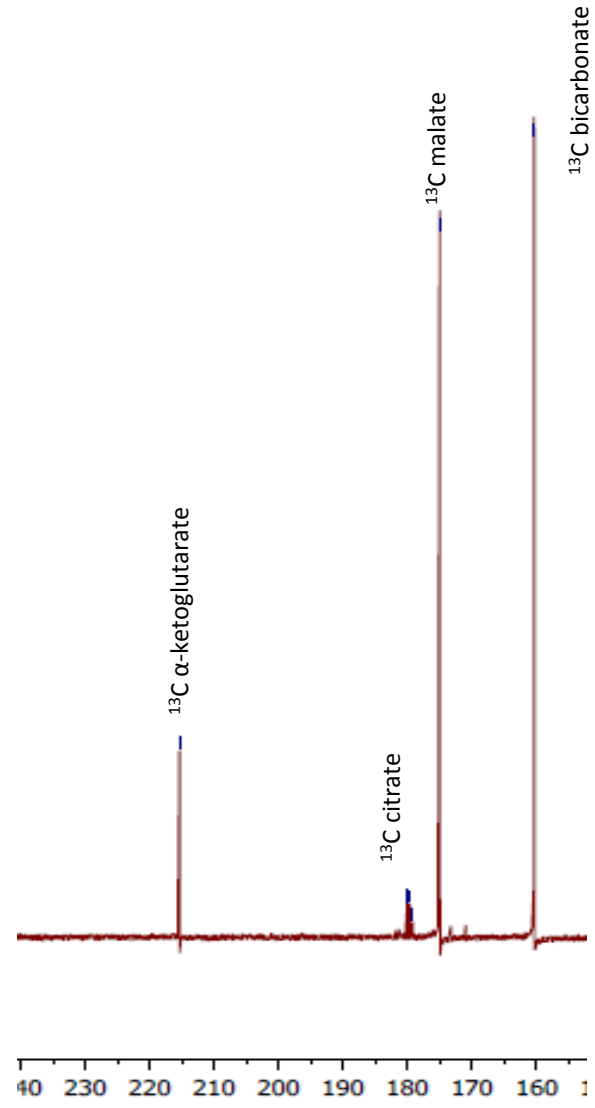

(b)

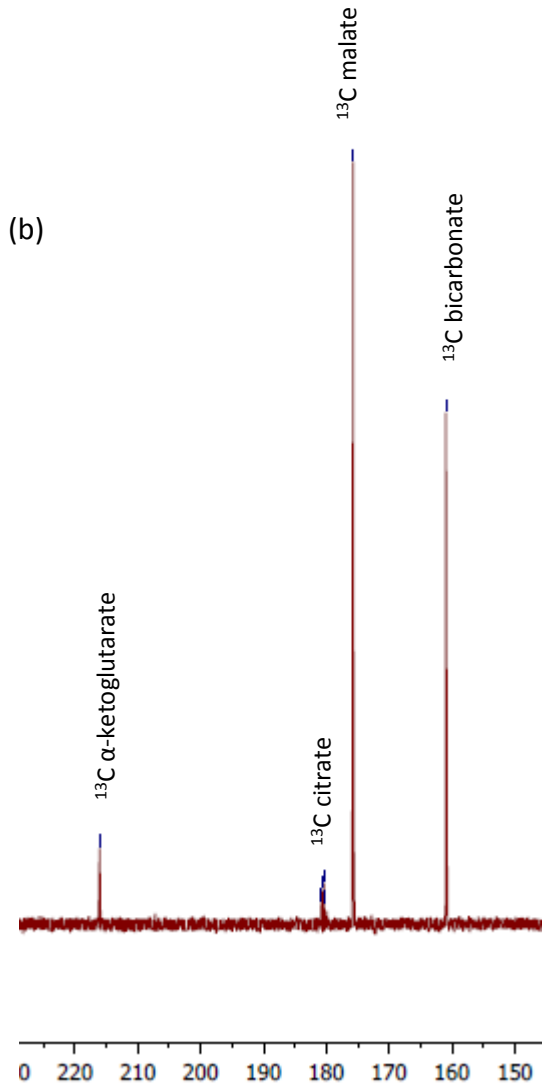

Supplement: FIGURE S2 — A section of the 13C spectra of (a) roots after 1 h, (b) roots after 2 h grown under low phosphate (5 μM P) conditions of V. divaricata. [file Data_Sheet_2.PDF]

(a)

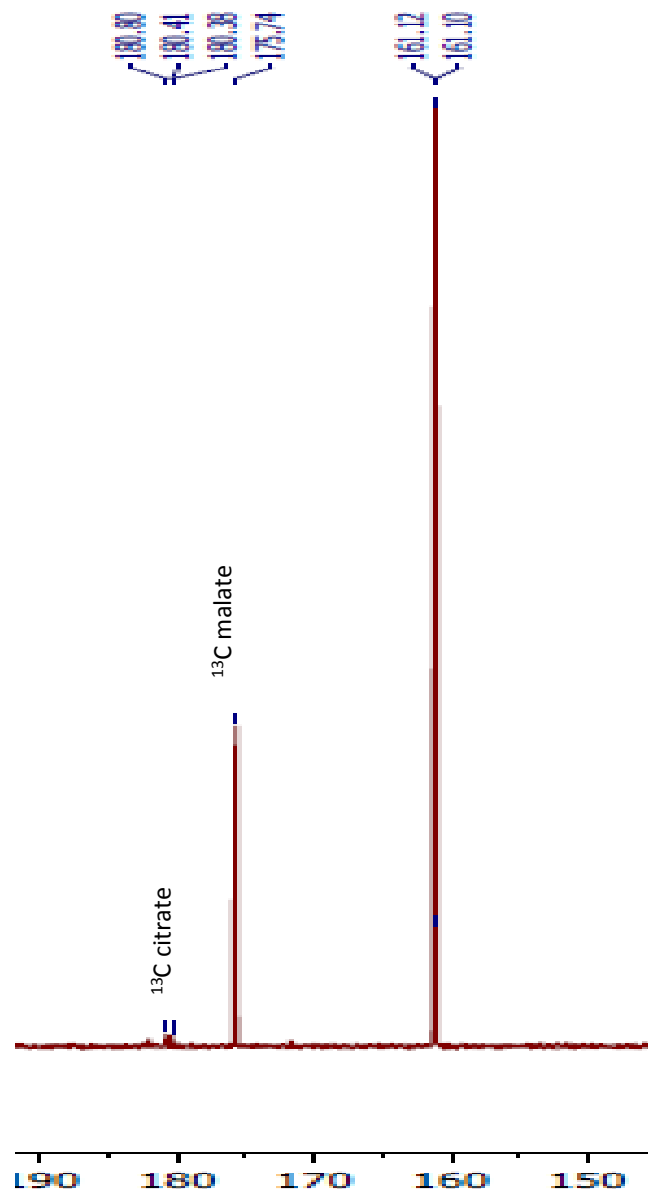

(b)

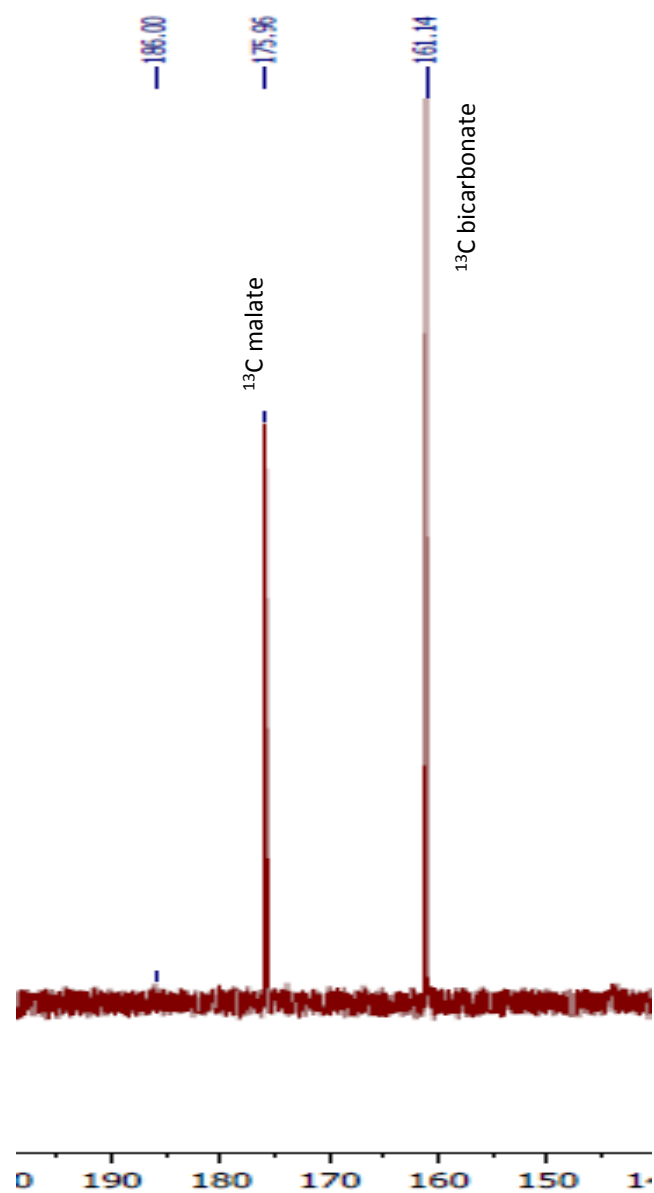

Supplement: FIGURE S3 — A section of the 13C spectra of (a) nodules after 1 h, (b) nodules after 2 h grown under high phosphate (500 μM P) conditions of V. divaricata. [file Data_Sheet_3.PDF]

(a)

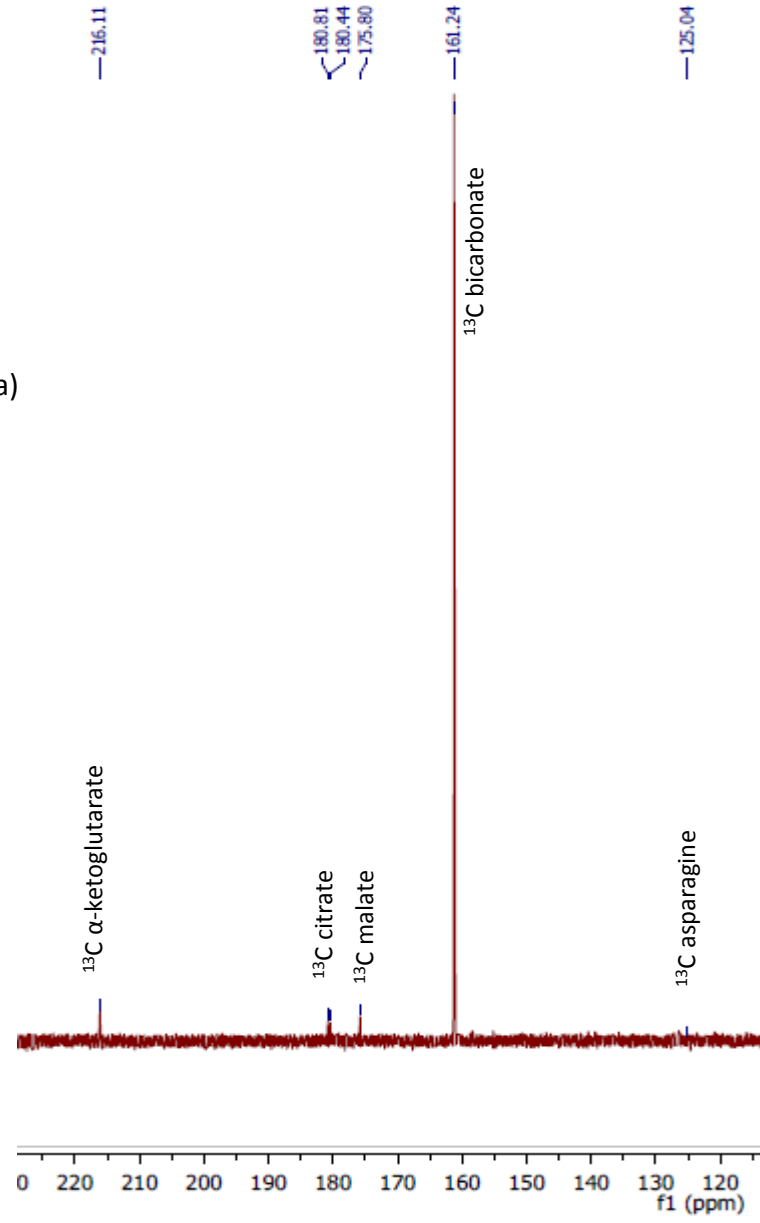

(b)

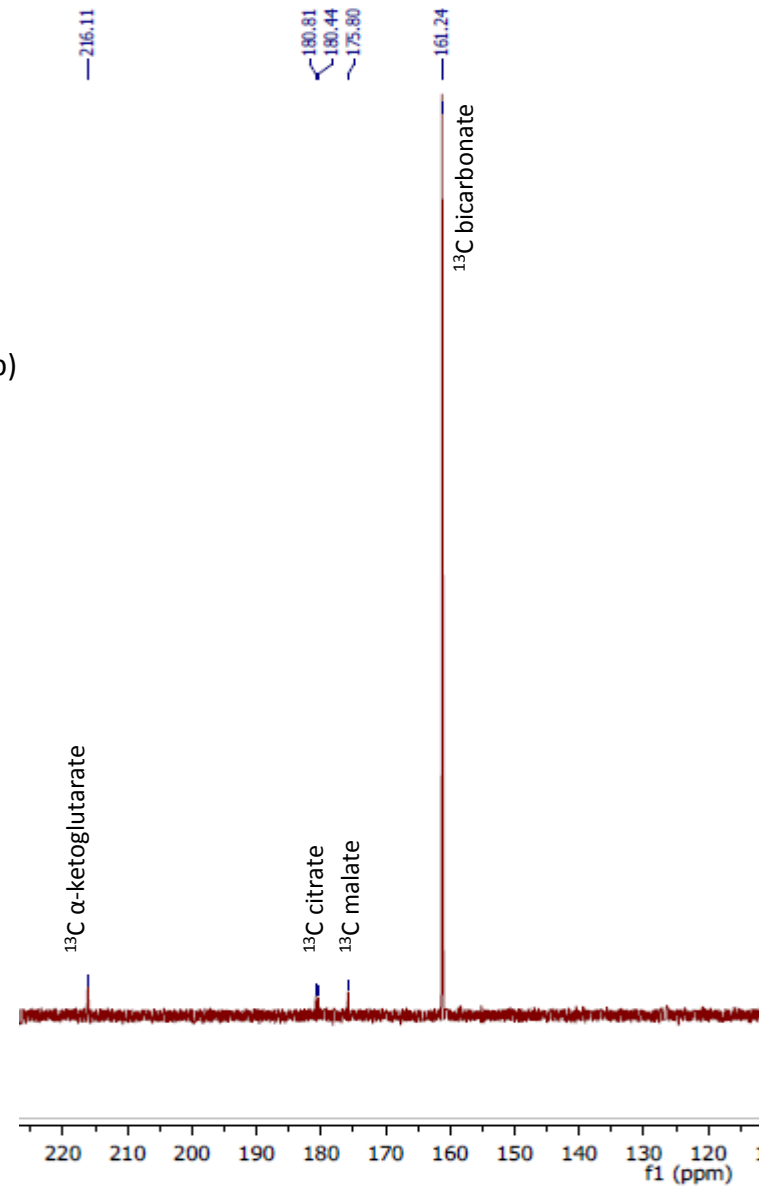

Supplement: FIGURE S4 — A section of the 13C spectra of (a) nodules after 1 h, (b) nodules after 2 h grown under low phosphate (5 μM P) conditions of V. divaricata. [file Data_Sheet_4.PDF]
