# Supplementary material for: Roots and Nodules Response Differently to P Starvation in the Mediterranean-Type Legume Virgilia divaricata
Source: Front Plant Sci. 2019 Feb 5;10:73. doi: 10.3389/fpls.2019.00073 (PMC6370976; doi:10.3389/fpls.2019.00073)

| | Parameter | Value |
|---|---|---|
| 1 | Data File Name | / GS015_13C_ref.fid / fid |
| 2 | Title | GS015_13C_ref |
| 3 | Comment | GS015 in D2O |
| 4 | Origin | Varian |
| 5 | Owner | |
| 6 | Site | |
| 7 | Instrument | inova |
| 8 | Author | |
| 9 | Solvent | d2o |
| 10 | Temperature | 25.0 |
| 11 | Pulse Sequence | s2pul |
| 12 | Experiment | 1D |
| 13 | Probe | BB_5mm |
| 14 | Number of Scans | 6288 |
| 15 | Receiver Gain | 54 |
| 16 | Relaxation Delay | 1.0000 |
| 17 | Pulse Width | 4.2500 |
| 18 | Presaturation Frequency | |
| 19 | Acquisition Time | 0.8688 |
| 20 | Acquisition Date | 2014-07-14T10:53:12 |
| 21 | Modification Date | 2014-07-14T14:13:22 |
| 22 | Class | |
| 23 | Spectrometer Frequency | 150.89 |
| 24 | Spectral Width | 47534.2 |
| 25 | Lowest Frequency | -2556.5 |
| 26 | Nucleus | 13C |
| 27 | Acquired Size | 41296 |
| 28 | Spectral Size | 131072 |

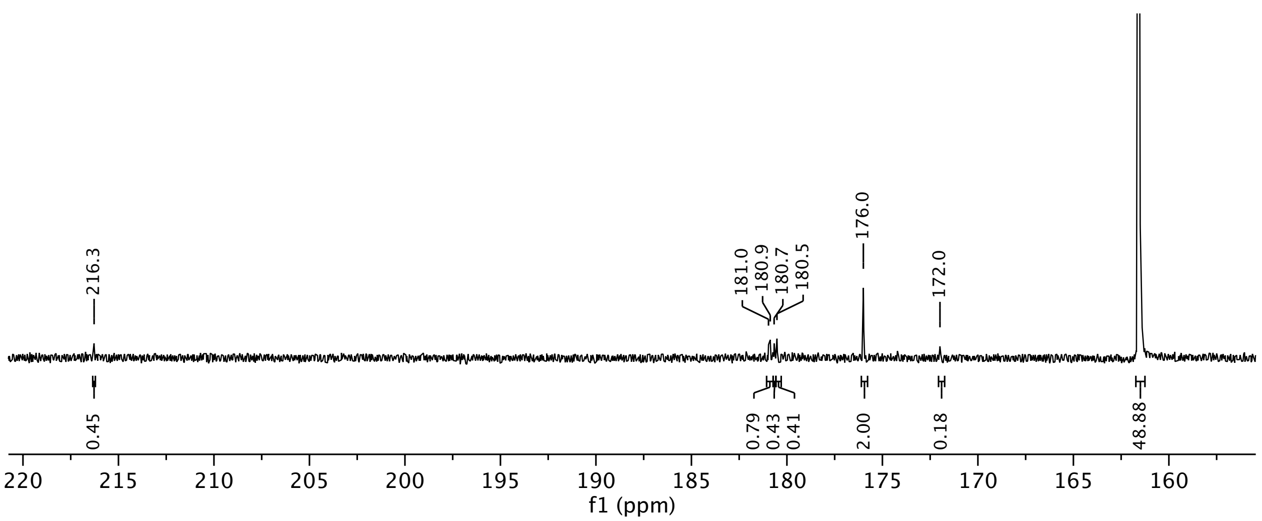


| | ppm | Area |
|---|---|---|
| 1 | 216.3 | 1827.36 |
| 2 | 181.0 | 1584.16 |
| 3 | 180.9 | 1568.36 |
| 4 | 180.7 | 1790.40 |
| 5 | 180.5 | 1957.12 |
| 6 | 176.0 | 10089.76 |
| 7 | 172.0 | 1841.80 |
| 8 | 161.6 | 128725.06 |
| 9 | 97.1 | 2663.26 |
| 10 | 83.5 | 1361.62 |
| 11 | 77.0 | 6704.11 |
| 12 | 75.4 | 2892.44 |
| 13 | 72.8 | 2633.02 |
| 14 | 72.6 | 5431.69 |
| 15 | 70.9 | 6793.56 |
| 16 | 70.6 | 1346.81 |
| 17 | 63.0 | 12426.06 |
| 18 | 62.2 | 2044.13 |
| 19 | 61.9 | 4006.08 |
| 20 | 59.7 | 9572.48 |
| 21 | 59.5 | 4323.61 |
| 22 | 58.9 | 10648.16 |
| 23 | 58.7 | 1190.95 |
| 24 | 53.9 | 2711.18 |
| 25 | 53.8 | 5035.95 |
| 26 | 53.0 | 10075.68 |
| 27 | 52.8 | 21550.29 |
| 28 | 52.0 | 16577.35 |
| 29 | 48.5 | 11278.90 |
| 30 | 31.4 | 7207.50 |
| 31 | 25.8 | 3044.19 |
| 32 | 25.5 | 4143.64 |
| 33 | 25.4 | 3911.53 |
| 34 | 25.2 | 9882.75 |
| 35 | 24.6 | 12927.74 |

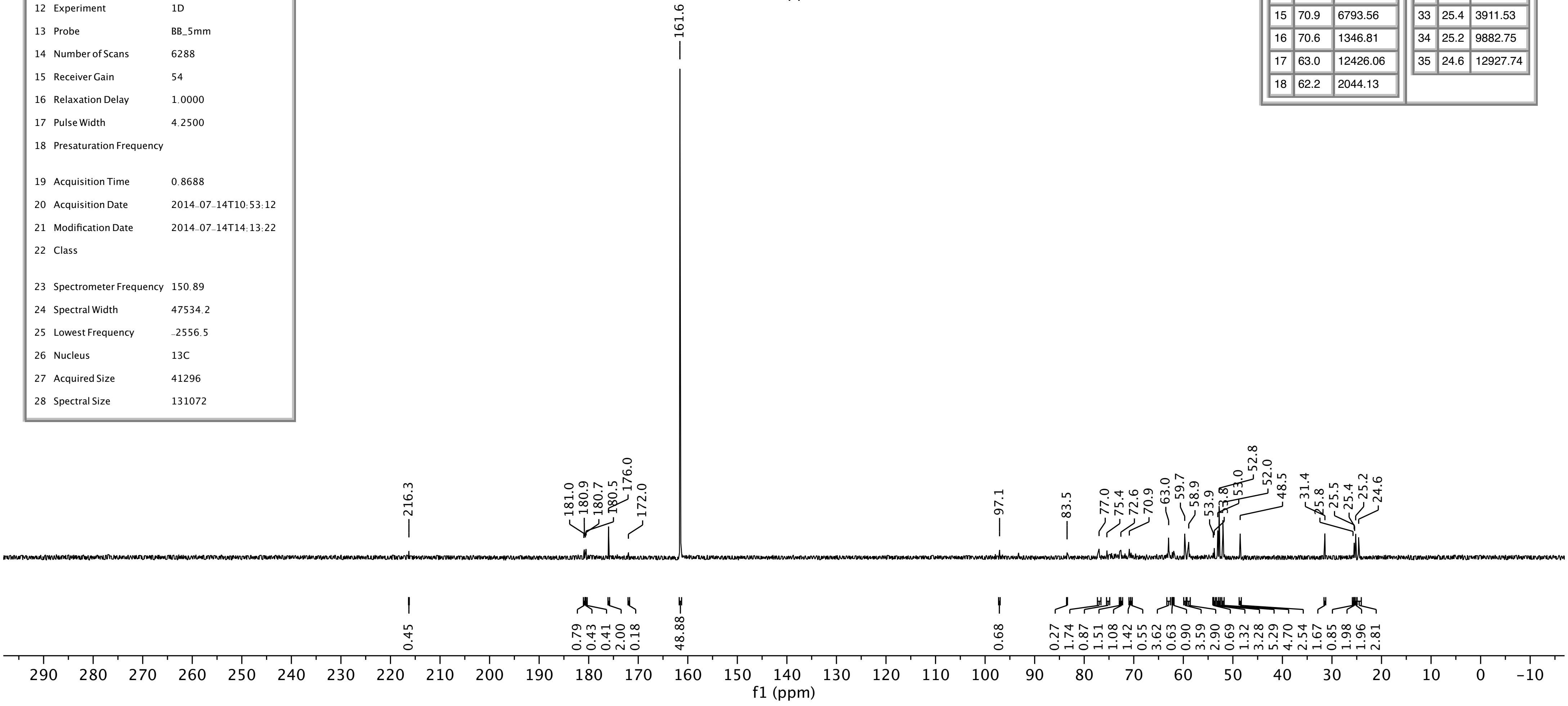

Supplement: FIGURE S5 — A sample of the full 13C spectra of roots after 1 h, from plants grown under high phosphate (500 μM P) conditions of V. divaricata. [file Data_Sheet_5.PDF]
