# Supplementary material for: Roots and Nodules Response Differently to P Starvation in the Mediterranean-Type Legume Virgilia divaricata
Source: Front Plant Sci. 2019 Feb 5;10:73. doi: 10.3389/fpls.2019.00073 (PMC6370976; doi:10.3389/fpls.2019.00073)

	Parameter	Value
1	Data File Name	GS009_C13_ref.fid / fid
2	Title	GS009_C13_ref
3	Comment	GS009 in D2O
4	Origin	Varian
5	Owner	
6	Site	
7	Instrument	inova
8	Author	vnmr1
9	Solvent	d2o
10	Temperature	25.0
11	Pulse Sequence	s2pul
12	Experiment	1D
13	Probe	dualbb
14	Number of Scans	2640
15	Receiver Gain	30
16	Relaxation Delay	1.0000
17	Pulse Width	4.6750
18	Presaturation Frequency	
19	Acquisition Time	1.3005
20	Acquisition Date	2014-04-29T16:39:37
21	Modification Date	2014-04-29T16:41:14
22	Class	
23	Spectrometer Frequency	100.57
24	Spectral Width	27653.0
25	Lowest Frequency	-1456.7
26	Nucleus	13C
27	Acquired Size	35962
28	Spectral Size	131072

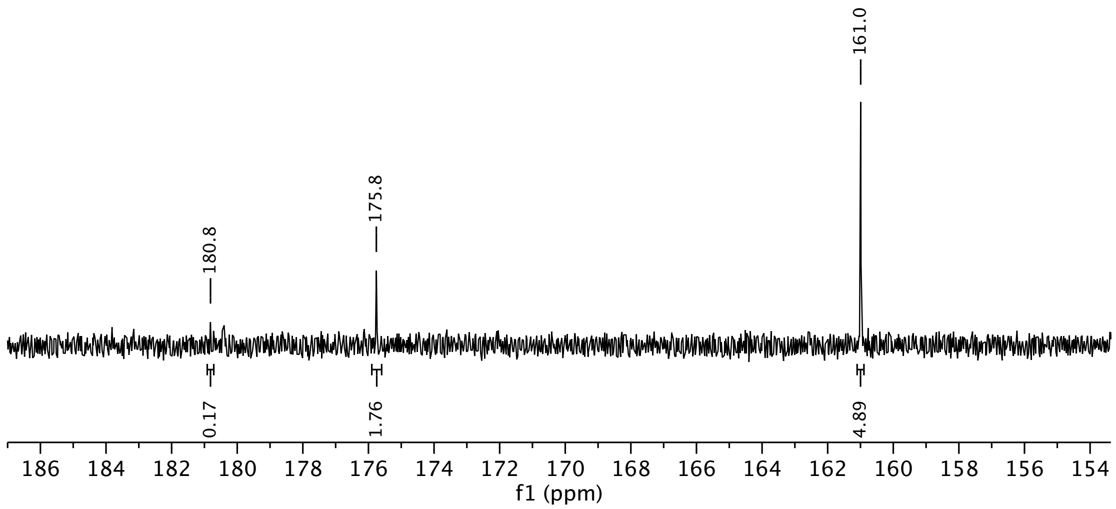

	ppm	Area
1	180.8	26.28
2	175.8	142.19
3	161.0	275.03
4	62.6	83.61
5	62.3	22.18
6	61.8	41.76
7	59.2	600.53
8	57.8	558.08
9	52.6	581.16
10	52.3	1182.14
11	51.2	1055.34
12	48.1	556.77
13	24.9	94.60
14	24.5	79.97

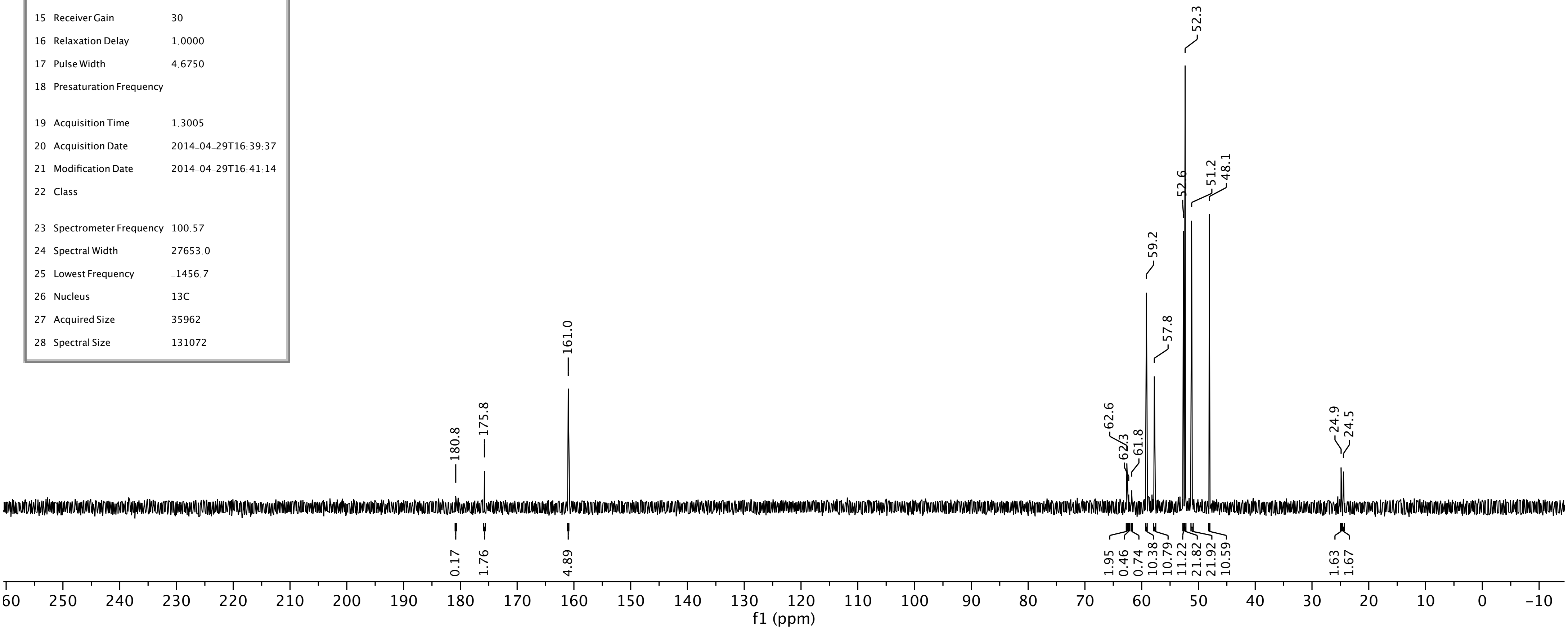

Supplement: FIGURE S6 — A sample of the full 13C spectra of nodules after 1 h, from plants grown under high phosphate (500 μM P) conditions of V. divaricata. [file Data_Sheet_6.PDF]
